# Numerical Study on Ultrasonic Guided Waves for the Inspection of Polygonal Drill Pipes

**DOI:** 10.3390/s19092128

**Published:** 2019-05-08

**Authors:** Xiang Wan, Xuhui Zhang, Hongwei Fan, Peter W. Tse, Ming Dong, Hongwei Ma

**Affiliations:** 1 School of Mechanical Engineering, Xi’an University of Science and Technology, Xi’an 710054, China; wx@xust.edu.cn (X.W.); fanhongwei84@163.com (H.F.); jesunatg@hotmail.com (M.D.); mahw@xust.edu.cn (H.M.); 2 Shaanxi Key Laboratory of Mine Mechanical and Electrical Equipment Intelligent Monitoring, School of Mechanical Engineering, Xi’an University of Science and Technology, Xi’an 710054, China; 3 Department of Systems Engineering and Engineering Management, City University of Hong Kong, Tat Chee Avenue, Kowloon 999077, Hong Kong, China; meptse@cityu.edu.hk

**Keywords:** ultrasonic guided waves, polygonal drill pipes, detection of damages

## Abstract

The polygonal drill pipe is one of the most critical yet weakest part in a high-torque drill machine. The inspection of a polygonal drill pipe to avoid its failure and thus to ensure safe operation of the drilling machine is of great importance. However, the current most frequently used ultrasonic inspection method is time-consuming and inefficient when dealing with a polygonal drill pipe, which is normally up to several meters. There is an urgent need to develop an efficient method to inspect polygonal drill pipes. In this paper, an ultrasonic guided wave technique is proposed to inspect polygonal drill pipes. Dispersion curves of polygonal drill pipes are firstly derived by using the semi-analytical finite element method. The ALID (absorbing layer using increasing damping) technique is applied to eliminate unwanted boundary reflections. The propagation characteristics of ultrasonic guided waves in normal, symmetrically damaged, and asymmetrically damaged polygonal drill pipes are studied. The results have shown that the ultrasonic guided wave technique is a promising and effective method for the inspection of polygonal drill pipes.

## 1. Introduction

Drilling machinery are important equipment in geological exploration, resource exploitation, ocean drilling, drilling in polar regions, and deep continental scientific drilling. The weakest part in a drilling machine is the drill stem. A drill stem is a critical component that links ground with underground. It functions by transferring torque and power to the drill bit underground, exerting bit pressure and circulating mud during the drilling. A drill stem is usually composed of a polygonal drill pipe, several circular drill pipes, a drill collar, a stabilizer, and several joints used to connect each part of the drill stem. A polygonal drill pipe is a special kind of drill pipe with a polygonal outer surface and circular inner surface. A polygonal drill pipe is connected between a rotary table and a circular drill pipe. Its main function is to turn the rotation of the rotary table into the rotation of the whole drill stem. During the drilling operation, a polygonal drill pipe suffers from harsh environments and complex loads. Therefore, defects (i.e., corrosion, cracks, and voids) are prone to occur in the interior of a polygonal drill pipe. The inspection of polygonal drill pipes is of great importance and urgency to ensure safe and reliable operation of drilling machines.

**Figure 1:**
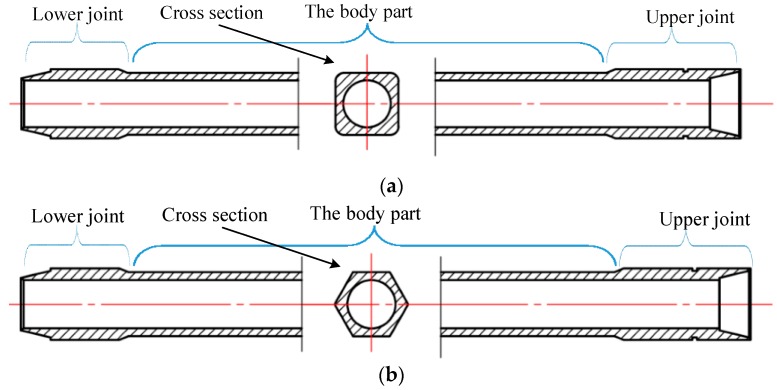
Structural diagram of polygonal drill pipes: (**a**) A square drill pipe; (**b**) a hexagonal drill pipe.

In practice, square and hexagonal drill pipes are two types of most frequently used polygonal drill pipes in high-torque drill machines. Typical square and hexagonal drill pipes are illustrated in Figure 1a,b, respectively. A polygonal drill pipe is composed of the upper joint, which is connected to a rotation table and the drill pipe body; and the lower joint, which is connected to a circular drill pipe. The length of a typical drill pipe body can be up to 10 meters. The cross section of a typical drill pipe body remains the same along the axis direction. Its outer surface is polygonal and its inner surface is circular. As shown in Figure 1a,b, the outer surfaces of the cross section of the square and hexagonal drill pipe body are square and hexagonal, respectively. The inner surfaces of the cross section are circular. We mainly focus on the polygonal drill pipe body, as the upper and lower joints in a polygonal drill pipe are quite short compared to the length of the drill pipe body. Consequently, in this paper, “a polygonal drill pipe” mainly refers to “the body part of a polygonal drill pipe”.

The ultrasonic technique is one of the most frequently used methods for inspecting drill pipes. Ushakov [1] reported the detection of cracks in drill pipes by using ultrasonic testing. An automatic ultrasonic flaw detection of the upset region of the drill pipe was proposed by Tu [2]. Chen [3] used ultrasonic phased array technology to detect the corrosion damage in the inner surface of drill pipes. In these studies, conventional ultrasonic technology was applied to inspect circular drill pipes. However, the inspection of polygonal drill pipes has received little attention, and very few studies have been conducted on the detection of damages in polygonal drill pipes.

In this study, we will focus on studying the inspection of polygonal drill pipes. The traditional ultrasonic methods are mainly based on point-to-point inspection systems, where the interrogating energy is conveyed in the form of shear or longitudinal bulk waves into a structure directly below the transmitter. In light of this fact, it is obvious that they become extremely time-consuming and inefficient when dealing with polygonal drill pipes with the length up to ten meters. Therefore, the conventional ultrasonic technique is not suitable for detecting damages in the polygonal drill pipes. There is an urgent need to develop an efficient method to inspect polygonal drill pipes.

The Ultrasonic guided wave technique [4,5,6,7,8] has recently evolved as a highly efficient inspection method for large-scale structures. It shows great potentials in NDT (Nondestructive Testing) for inspecting structures of many fields (i.e., plates [9,10,11,12], pipelines [13,14,15,16], and railways [17]). Ultrasonic guided waves enable a line-to-line inspection method, which makes it uniquely suitable for inspecting large structures. It is expected to alleviate the aforementioned disadvantages and to improve the inspection efficiency. According to our literature review, using ultrasonic guided waves for the detection defects in polygonal drill pipes has not been reported. In this study, the ultrasonic guided wave technique is proposed to inspect the long length of polygonal drill pipes. With this purpose, two major issues will be investigated and addressed. First, characteristics of ultrasonic guided waves propagating in polygonal drill pipes are studied. Their phase and group velocity dispersion curves are derived. Proper wave modes are identified and used to inspect polygonal drill pipes. Second, ultrasonic guided waves with the identified modes are used to inspect polygonal drill pipes. The interaction of ultrasonic guided waves with symmetric and asymmetric damages in polygonal drill pipes are also discussed. During the numerical simulation studies, ALID (absorbing layer using increasing damping) is introduced to both ends of polygonal drill pipe models to eliminate unwanted boundary reflections. The rest of this paper is organized as follows. The SAFE (semi-analytical finite element method) is used to study the dispersion relations of polygonal drill pipes in Section 2. Numerical setups are presented in Section 3, and ALID is also introduced in this section. Numerical results are shown and discussed in Section 4. Conclusions are drawn, at last, in Section 5.

## 2. Characteristics of Guided Waves Propagating in Polygonal Drill Pipes

When applying ultrasonic guided waves to inspect polygonal drill pipes, knowing about the characteristics of ultrasonic guided waves propagating in polygonal drill pipes is quite necessary. Obtaining their dispersive relations is a prerequisite. Due to the complex geometry of the cross section, building the analytical dispersion equation is quite difficult. In this section, the SAFE method [18,19,20,21] was proposed to study characteristics of guided waves travelling in polygonal drill pipes and to derive their dispersion curves.

### 2.1. SAFE Formulations

In this subsection, a square drill pipe was taken as an example to illustrate how the SAFE method is employed to derive its phase and group velocity dispersion curves.

**Figure 2:**
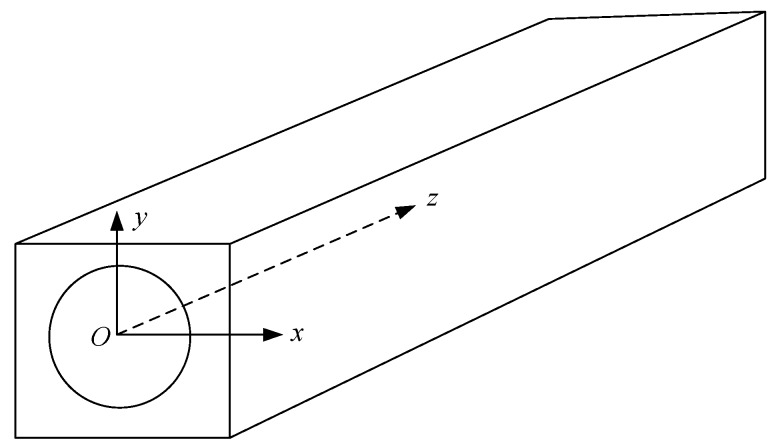
SAFE (semi-analytical finite element) model of a square drill pipe.

Assuming the cross section of a square drill pipe is x–y plane and waves propagate in the z-direction as shown in Figure 2.

The displacement, strain, and stress vectors at any point (*x, y, z*) in the media of a square drill pipe are denoted as **u**, **ε**, and **σ** respectively. The strain–stress relationship is expressed as σ=Cε, where C is the elastic stiffness tensor. The strain vector is written by the strain–displacement relationship as

(1) $$\varepsilon =\left[L_{x}\frac{\partial }{\partial x}+L_{y}\frac{\partial }{\partial y}+L_{z}\frac{\partial }{\partial y}\right]u,$$

where the formulations of *L*_x_*, L*_y_*,* and *L*_z_ can be found in the references [20,21].

The displacement field is assumed to be harmonic, propagating along the direction z, and spatial functions are used to describe its amplitude in the cross-sectional plane *x*–*y* as

(2) $$u\left(x,y,z,t\right)=\left[\begin{array}{c} u_{x}\left(x,y,z,t\right)\\ u_{y}\left(x,y,z,t\right)\\ u_{z}\left(x,y,z,t\right) \end{array}\right]=\left[\begin{array}{c} u_{x}\left(x,y\right)\\ u_{y}\left(x,y\right)\\ u_{z}\left(x,y\right) \end{array}\right]e^{i\left(\xi z-\omega t\right)},$$

where *i* is the imaginary unit, ξ is the wave number, and ω denotes the angular frequency.

The square drill pipe’s cross-sectional domain Ω can be represented by a system of finite elements with domain $\Omega_{e}$. The discretised version of the displacement expressions in Equation (2) over the element domain can be expressed in terms of the shape functions $N_{k}\left(x,y\right)$, and the nodal unknown displacements ($U_{xk},U_{yk},U_{zk}$) in the *x*, *y*, and *z* directions as

(3) $$u^{\left(e\right)}\left(x,y,z,t\right)={\left[\begin{array}{c} \sum_{k=1}^{n}N_{k}\left(x,y\right)U_{xk}\\ \sum_{k=1}^{n}N_{k}\left(x,y\right)U_{yk}\\ \sum_{k=1}^{n}N_{k}\left(x,y\right)U_{zk} \end{array}\right]}^{\left(e\right)}e^{i\left(\xi z-\omega t\right)}=N\left(x,y\right)q^{\left(e\right)}e^{i\left(\xi z-\omega t\right)},$$

where *n* denotes the number of nodes per element, and N(x,y) and $q^{\left(e\right)}$ are shape function matrix and nodal displacement vector, respectively. Their expressoion can be found in the references [20,21].

The strain vector in the element represented as a function of the nodal displacements are written as

(4) $$\varepsilon^{\left(e\right)}=\left[L_{x}\frac{\partial }{\partial x}+L_{y}\frac{\partial }{\partial y}+L_{z}\frac{\partial }{\partial y}\right]N\left(x,y\right)q^{\left(e\right)}e^{i\left(\xi z-\omega t\right)}=\left(B_{1}+i\xi B_{2}\right)q^{\left(e\right)}e^{i\left(\xi z-\omega t\right)},$$

(5) $$B_{1}=L_{x}N_{,x}+L_{y}N_{,y},$$

(6) $$B_{2}=L_{z}N,$$

where $N_{,x}$ and $N_{,y}$ are the derivatives of the shape function matrix with respect to the *x* and *y* directions, respectively.

The homogeneous general wave equation is obtained as [18]

(7) $${\left[K_{1}+i\xi K_{2}+\xi^{2}K_{3}-\omega^{2}M\right]}_{M}U=0,$$

where the formulations of $K_{1}$, $K_{2}$, $K_{3}$, and M are illustrated in the references [20,21].

The final form of the eigenvalue problem in Equation (7) is written as

(8) $${\left[K_{1}+\xi {\hat{K}}_{2}+\xi^{2}K_{3}-\omega^{2}M\right]}_{M}\hat{U}=0,$$

where $\hat{U}$ is a new nodal displacement vector. Nontrivial solutions can be obtained by solving a twin-parameter generalized eigenproblem in wave number ξ and angular frequency and ω.

For a given value of ξ, the corresponding value of ω can be obtained by solving the eigenvalue problem of Equation (8). By applying $c_{p}=\omega /\xi$, the phase velocity dispersion curves can be derived.

Group velocity can be calculated directly at each (ξ,ω) solution point without any contribution from adjacent points. The procedure starts by evaluating the derivative of the Equation (8) with respect to the wavenumber ξ:

(9) $$\frac{\partial }{\partial \xi }\left(\left[K\left(\xi \right)-\omega^{2}M\right]{\hat{U}}_{R}\right)=0,$$

where $K\left(\xi \right)=K_{1}+\xi {\hat{K}}_{2}+\xi^{2}K_{3}$ and ${\hat{U}}_{R}$ denotes the right eigenvector. Pre-multiplying the Equation (18) by the transpose of the left eigenvector ${\hat{U}}_{L}^{T}$:

(10) $${\hat{U}}_{L}^{T}\left[\frac{\partial }{\partial \xi }K\left(\xi \right)-2\omega \frac{\partial \omega }{\partial \xi }M\right]{\hat{U}}_{R}=0.$$

Since ∂ω/∂ξ is a scalar, the group velocity can be expressed as

(11) $$c_{g}=\frac{\partial \omega }{\partial \xi }=\frac{{\hat{U}}_{L}^{T}\left({\hat{K}}_{2}+2\xi K_{3}\right){\hat{U}}_{R}}{2\omega {\hat{U}}_{L}^{T}M{\hat{U}}_{R}}.$$

From the Equation (11), the group velocity can be computed for each individual solution (ξ,ω) of the dispersion relations at a time independently of any adjacent solution.

### 2.2. Phase and Group Velocity Dispersion Curves in Polygonal Drill Pipes

**Figure 3:**
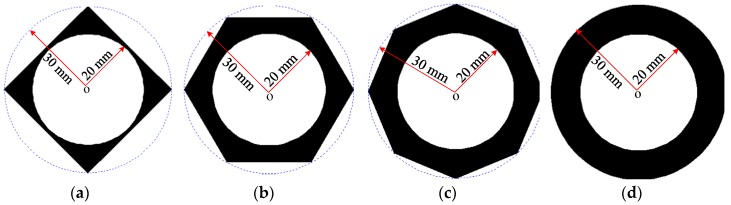
Cross sections: (**a**) A square drill pipe; (**b**) a hexagonal drill pipe; (**c**) an octagonal drill pipe; (**d**) the referential hollow cylinder.

By using the formulations in Section 2.1, phase and group velocity dispersion curves in polygonal drill pipes could be derived. In this section, square, hexagonal, and octagonal drill pipes, as well as a referential hollow cylinder were considered. Their cross sections are shown in Figure 3. The inner surfaces of polygonal drill pipes are circle with a radius of 20 mm, which is equal to the inner radius of the referential hollow cylinder. The outer surfaces of polygonal drill pipes are square, hexagon, and octagon, respectively. The radius of their circumcircle is 30 mm, which is identical to the outer radius of the referential hollow cylinder. The dispersion curves of the referential hollow cylinder have been known for a long time and are used as references to study the dispersion characteristics of polygonal drill pipes.

**Figure 4:**
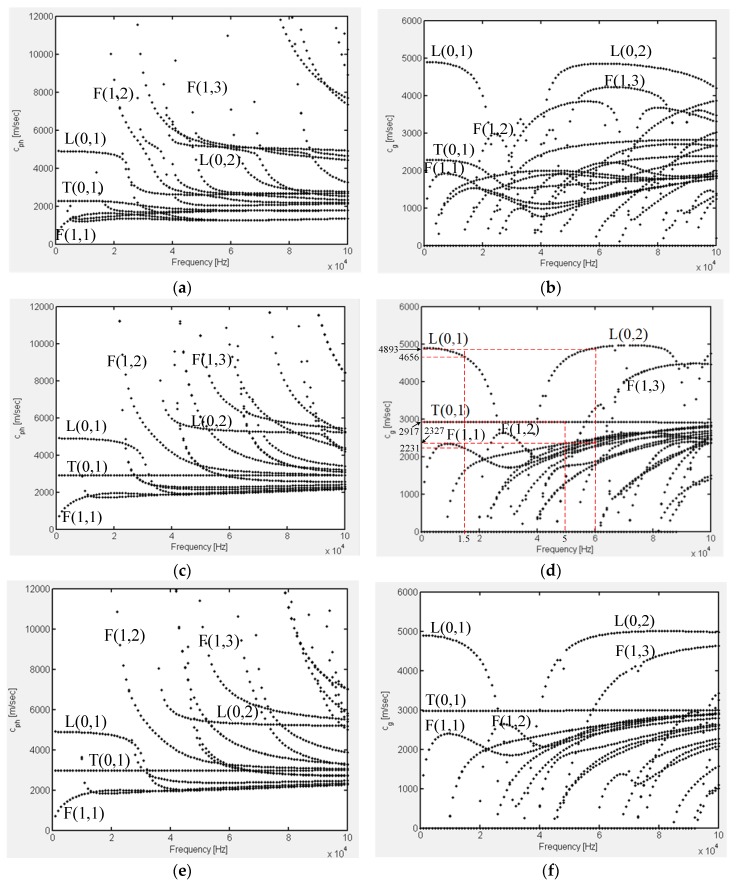
Phase velocity dispersion curves of: (**a**) a square drill pipe, (**c**) a hexagonal drill pipe, and (**e**) an octagonal drill pipe; and group velocity dispersion curves of: (**b**) a square drill pipe, (**d**) a hexagonal drill pipe, and (**f**) an octagonal drill pipe.

**Figure 5:**
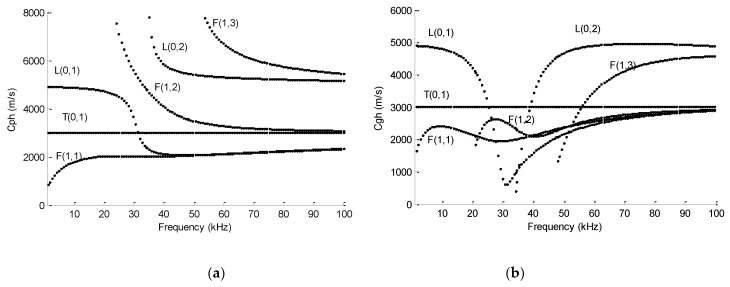
Dispersion curves of the referential hollow cylinder with the inner radius of 20 mm and the thickness of 10 mm: (**a**) Phase velocity dispersion curves; and (**b**) group velocity dispersion curves.

The material of polygonal drill pipes, as well as the referential hollow cylinder, is carbon steel. The density, elastic modulus, and Poisson’s rate are 8000 kg/m^3^, 192 GPa, and 0.33, respectively. Phase velocity and group velocity dispersion curves of polygonal drill pipes can be derived by the GUIGUW software [22]. Figure 4a,c,e refer to the phase velocity dispersion curves of the square, hexagonal, and octagonal drill pipes, respectively. Figure 4b,d,f illustrate the corresponding group velocity dispersion curves. Dispersion curves of symmetric modes of the referential hollow cylinder are illustrated in Figure 5. From these figures, several findings can be clearly observed. First, for each polygonal drill pipe, at a specific frequency, there are multiple modes in existence and the number of modes is much larger than that of the referential hollow cylinder. Second, the number of modes is increased with the frequency. Third, as the number of edges of the outer surface of the cross section is increased, the phase and group velocity dispersion curves become sparse.

**Figure 6:**
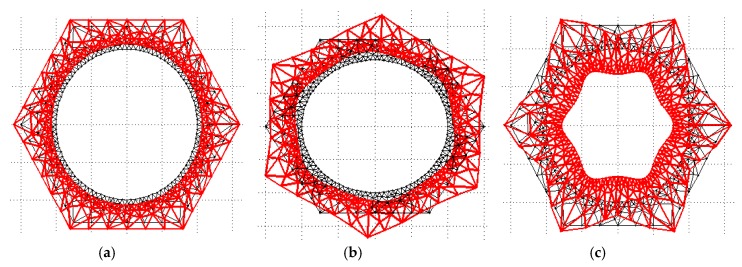
Hexagonal drill pipe’s mode shape: (**a**) L(0,1) mode; (**b**) T(0,1) mode; and (**c**) L(0,2) mode.

Each Lamb wave mode in polygonal drill pipes can be identified by its wave shape. First order longitudinal L(0,1) and torsional T(0,1) modes, as well as second order longitudinal mode L(0,2) in these polygonal drill pipes are identified. These identified modes are labelled in the corresponding figures. Hexagonal drill pipe’s mode shapes of the first order longitudinal and torsional, as well as second order longitudinal modes are illustrated in Figure 6a–c, respectively. As the edges of the cross section is increased, dispersion curves of L(0,1), T(0,1), and L(0,2) modes are getting close to those of the referential hollow cylinder. L(0,1) mode at the frequency below 20 kHz and L(0,2) mode at the frequency range from 40 to 100 kHz are almost flat and possess the highest group velocity. The nearly flat characteristic of the dispersion curves indicates that these two modes are almost non-dispersive and they can propagate a very long distance with the duration of the wave packet unchanged. These two modes with the highest group velocity travel faster than any other modes. Therefore, they will appear ahead in the time domain waveform, which will make the received waveform much more convenient to process and interpret. The dispersion curve of T(0,1) mode in the square drill pipe is not a straight line yet. However, the dispersion curves of T(0,1) mode in the hexagonal and octagonal drill pipes are straight, which are similar to that of the referential hollow cylinder. T(0,1) mode is also non-dispersive, making it the preferred candidate for inspecting polygonal drill pipes. Therefore, L(0,1), L(0,2), and T(0,1) modes can be used for inspecting polygonal drill pipes.

## 3. Numerical Setups

The hexagonal drill pipe is one of the most frequently used polygonal drill pipe, thus it was taken as an example to study ultrasonic guided waves for the inspection of polygonal drill pipes. In order to eliminate the influence of boundary reflections, ALID (absorbing layer using increasing damping) was added to finite element models of hexagonal drill pipes. It is firstly introduced in Section 3.1.

### 3.1. The ALID Technique

The ALID is an absorbing layer that is made of a material with the same properties as those of the area of study, except for having a gradually increasing damping.

The equation of dynamic equilibrium in the time domain is written as

(12) $$\left[M\right]\ddot{u}+\left[C\right]\dot{u}+\left[K\right]u=f,$$

with [M], [C], and [K] referring to the mass, damping, and stiffness matrices, respectively.

Stiffness or mass proportional damping can be introduced in time domain finite models and it is generally termed as Rayleigh damping. Consequently, the damping matrix [C] can be expressed as

(13) [C]=α[M]+β[K],

where α and β denote the mass and stiffness proportional damping coefficients.

In an ALID with a boundary perpendicular to the x axis, the value of α and β are gradually increased in the x direction. The following formulations are set:

(14) $$\alpha \left(x\right)=\alpha_{\max}X{\left(x\right)}^{m}\;\;and\;\;\;\beta \left(x\right)=\beta_{\max}X{\left(x\right)}^{m},$$

where $\alpha_{\max}$ and $\beta_{\max}$ are positive real numbers and X(x) varies from 0 at the interface between the ALID and the area of study to 1 at the end of the ALID, following a power law whose order is defined by *m*.

It is noted that the introduction of damping decreases with the value of the stable time increment when solving the finite element model with central difference explicit scheme [18]. The damping value at the end of an ALID is usually very large compared to the values commonly used in the structures. A high value of α causes a relatively small decrease in the stable increment, whereas a value of β usually has a very strong effect leading to a great loss in computational efficiency. Therefore, it is preferable to avoid using β to define ALID with an explicit scheme. In this paper, we only have α for numerical studies. The Equation (14) changes to the following formulation

(15) $$\alpha \left(x\right)=\alpha_{\max}X{\left(x\right)}^{m}\;\;and\;\;\;\;\;\beta \left(x\right)=0.$$

It is obvious to see that the proper definition of the layer parameters (i.e., the length of the layer *L*), variation of the attenuation parameter α, and the power law *m* is essential to achieve an efficient and accurate model. In FE (Finite element) models, as the space is discretized, the gradual increase of α occurs by steps. An ALID is defined as a series of sub layers having the same material properties but different values of α. It is preferable to minimize the change of α between two adjacent sub layers. It is recommended to have one-element-thick sub layers.

Suppose an ALID with the length *L_a_* has *n* sub layers, and the length of each sub layer is *l_a_*, the attenuation parameter of *i*th sub layer α(i) is defined as.

(16) $$\alpha \left(i\right)=\alpha_{\max}{\left(\frac{il_{a}}{L_{a}}\right)}^{m},$$

where *i* varies from 1 to *n*. The value of *i* equals to 1, corresponding to the sub layer next to the interface and *n* corresponding to the sub layer at the end of the ALID. According to [23], in order to achieve an efficient and accurate model, these parameters can be selected as follows. $\alpha_{\max}$ is selected to be larger than 10 *f*_0_, where *f*_0_ denotes the excitation frequency. *L_a_* is set to larger than 2λ, in which λ refers to the wavelength. The length of the sub layer *l_a_* equals to element size. The value of *m* is set to 2 or 3.

### 3.2. FE Model

**Figure 7:**
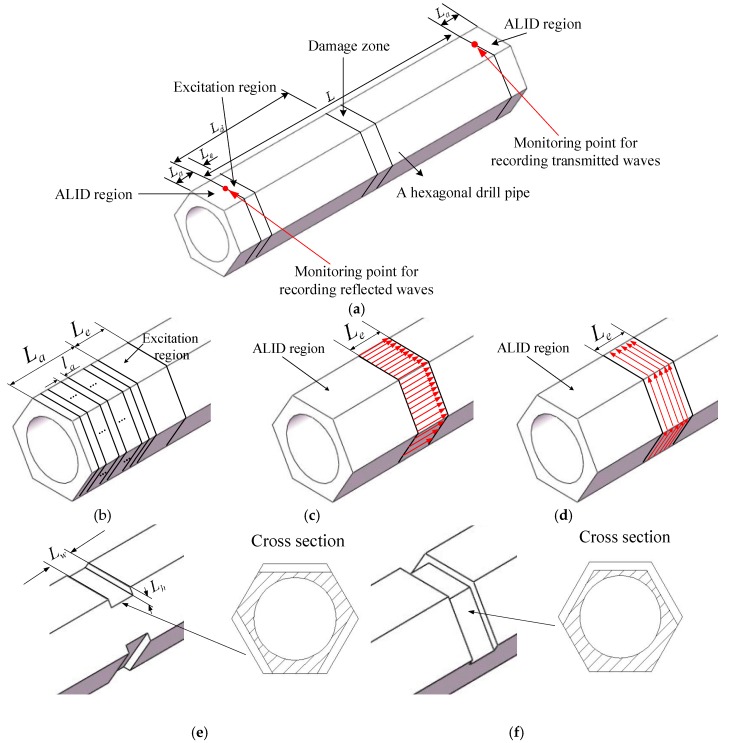
Schematic model for the hexagonal drill pipe: (**a**) Overall finite element model; (**b**) ALID (absorbing layer using increasing damping) model; (**c**) excitation for generating longitudinal modes; (**d**) excitation for generating torsional modes; (**e**) symmetric damage model; and (**f**) asymmetric damage model.

In this paper, numerical simulations were performed by using ABAQUS software. A schematic finite element model for the hexagonal drill pipe is illustrated in Figure 7a. The radius of the circular inner surface of the hexagonal drill pipe is 20 mm. The circumcircle radius of the outer surface of the hexagonal drill pipe is 30 mm. The length of the hexagonal drill pipe is *L*. ALID regions are applied at both ends with a length of *L_a_*. The detailed information for the ALID regions is shown in Figure 7b. The ALID region is consisted of a series of sub layers with a length of *l_a_*. Uniform surface traction is symmetrically enforced on the outer surface of the excitation region which is located at the left end of the hexagonal drill pipe. The length of the excitation region is *L_e_*. As shown in Figure 7c,d, axial and circumferential surface tractions are distributed uniformly and symmetrically around the outer surface of the excitation region to generate longitudinal and torsional modes in the hexagonal drill pipe, respectively. The damage zone is located at a position *L_d_* from the left end of the hexagonal drill pipe. For the normal hexagonal drill pipes, there is no damage in the damage zone. For symmetrically damaged hexagonal drill pipe, as illustrated in Figure 7e, there is a symmetric damage which is consisted of three slots symmetrically distributed around the damage zone. The length and the height of the slots are denoted by *L_w_* and *L_h_*, respectively. Similarly, for the asymmetrically damaged hexagonal drill pipe, as shown in Figure 7f, there is an asymmetric defect which consisted of three adjacent slots. Two monitoring points marked by red dots are located at the center of the left and right edges of the hexagonal drill pipe. They are used to record reflected and transmitted time domain waveforms, respectively. It is noted that for longitudinal modes (i.e., L(0,1) and L(0,2) modes) that axial displacements are recorded, and for torsional modes (i.e., T(0,1) mode), circumferential displacements are recorded.

### 3.3. Excitation Signal

A tone burst consisting of *N* cycles with a specified center frequency *f*_0_ was used as the excitation signal and it is formulated in Equation (17)

(17) $$F(t)=F_{0}\sin\left(2\pi f_{0}t\right)\times {\left(\sin\left(\pi f_{0}t/N\right)\right)}^{2},$$

where *F_0_* refers to the amplitude of the excited signal.

**Figure 8:**
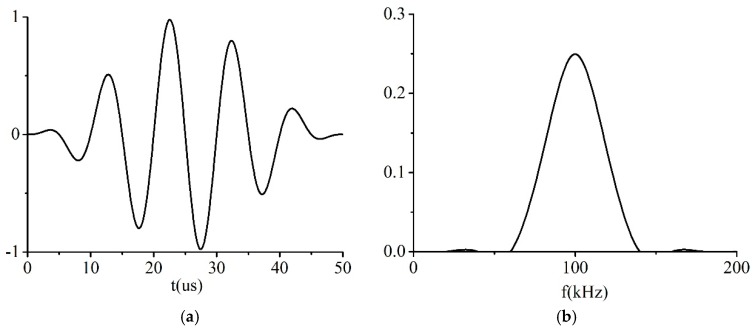
Excitation signal: (**a**) Temporal waveform; and (**b**) frequency spectrum.

An example of the excitation temporal waveform and its frequency spectrums are illustrated in Figure 8a,b, respectively.

### 3.4. Element Size and Time Step

In general, a higher-order element type, a denser mesh, and smaller time step will give a more accurate simulation result, but will also cost more in terms of calculation time and computer resources. In order to obtain adequate accuracy and high efficiency, in normal polygonal drill pipes, a second-order rectangular element type was used for discretization and the maximum element size and time step was adopted according to [24,25]. For the numerical integration scheme, the explicit method was used. The integration method was the central difference method

(18) $$\Delta I=\frac{\lambda_{\min}}{20},$$

(19) $$\Delta t=\frac{1}{20f_{\max}},$$

where ΔI is the element size and Δt the time step; $\lambda_{\min}$ and $f_{\max}$ are shortest wavelength and highest frequency of interest, respectively.

**Figure 9:**
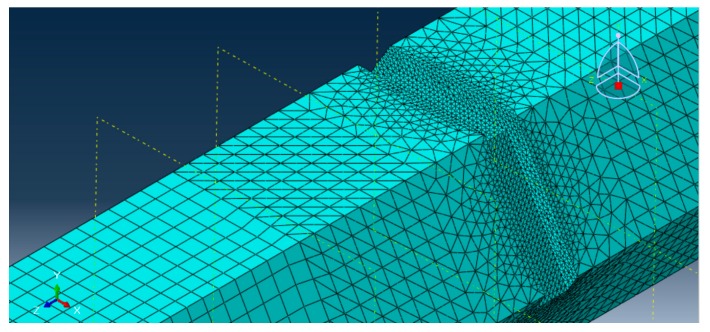
An example of meshing results for a hexagonal drill pipe with an asymmetric damage.

For a polygonal drill pipe with a damage, in the damage region, the element size was selected smaller than in the normal region. An example of meshing results is illustrated in Figure 9.

## 4. Results and Discussions

In this section, three important influential factors on the ultrasonic guided waves propagating in polygonal drill pipes are studied in Section 4.1. They are the selection of the proper number of excitation burst cycles, the temporal waveforms received from polygonal drill pipes with the outer surfaces of increasing number of edges, and the effectiveness of the applied ALIDs. Ultrasonic guided waves of longitudinal and torsional modes interacting with symmetric and asymmetric damages in polygonal drill pipes are investigated in Section 4.2.

### 4.1. Influential Factors on the Ultrasonic Guided Waves Propagating in Polygonal Drill Pipes

#### 4.1.1. The Influence of the Number of Excitation Burst Cycles on Received Temporal Waveforms

The tone-burst signal for exciting guided waves in a waveguide is normally a few cycles of sine waves modulated by the Hanning window. The larger the number of the excitation signal, the wider the duration of the excitation pulse, and the narrower the band of its frequency spectrum. The selection of a proper number of excitation burst cycles is a perquisite when using ultrasonic guided waves for non-destructive testing. When selecting a small number, unwanted modes, which lie close the desired mode, may be generated simultaneously, especially for complex waveguides whose dispersion curves are dense (e.g. polygonal drill pipes). When selecting a large number, the desired wave packet (i.e., the reflected waveform from a damage) may overlap with unwanted wave packets (e.g., the reflections from edges). Therefore, selecting a proper number of excitation burst cycles is of great importance when inspecting polygonal drill pipes by ultrasonic guided waves.

**Figure 10:**
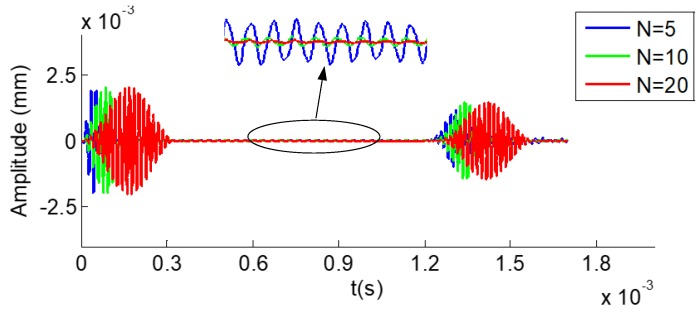
Time domain waveform received from a hexagonal drill pipe under the excitation of tone burst with different cycles.

Figure 10 illustrates the received time–domain waveforms received from a hexagonal drill pipe under the excitation of the tone burst pulse with 5, 10, and 20 cycles, denoted by blue, green, and red curves, respectively. The middle upper part of the figure shows the corresponding local enlargement. The length of the polygonal drill pipe was 3000 mm. Axial surface traction was exerted on the left section. Longitudinal modes at the center frequency of 60 kHz were excited. The received point was also set at the left edge. In the figure, the first and second wave packets refer to the direct and reflected waves, respectively. The propagation distance of the reflected wave packets was 6000 mm. They appeared at around 1.24 × 10^−3^ s. The group velocity was calculated as 6000 mm/1.24 × 10^−3^ s = 4838 m/s, which is close to the theoretical group velocity of L(0,2) mode shown in Figure 4. It was verified that the reflected wave packets were L(0,2) mode. It was clearly observed that the waveform generated from the tone burst of five cycles was prone to suffer from the fluctuation. It was inferred that the fluctuation may have resulted from the influence of the adjacent modes of the desired mode. The fluctuation contributed to the poor signal-to-noise ratio of the waveform of five cycles. However, the signal-to-noise ratio was improved significantly in the received waveforms of 10 and 20 cycles. Furthermore, it was found that the signal-to-noise ratio was increased with the number of cycles of the excitation tone burst. In this paper, a tone burst of 10 or 20 cycles were used for the excitation of ultrasonic guided waves in polygonal drill pipes.

#### 4.1.2. The Influence of the Outer Surfaces of Increasing Edges on Temporal Signals

In this subsection, time domain responses of square, hexagonal, and octagonal drill pipes and the referential pipe under the same excitation conditions are compared and studied. Their sectional parameters are the same as illustrated in Figure 3a–d, respectively. The length of the polygonal drill pipes and the referential pipe were set to 3000 mm. Longitudinal L(0,2) mode at the center frequency of 60 kHz was excited in these waveguides.

**Figure 11:**
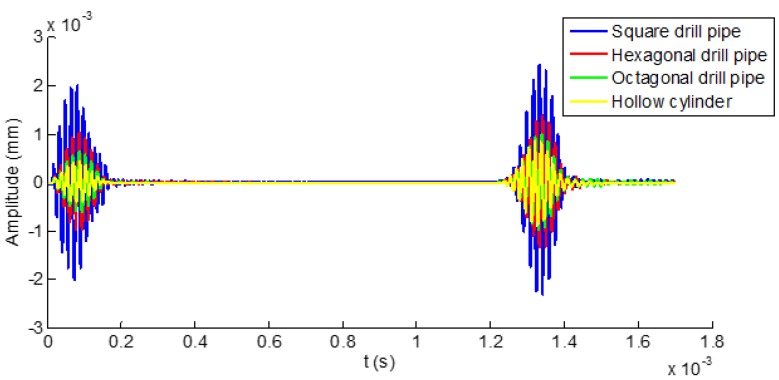
Time domain waveforms received from square, hexagonal, and octagonal drill pipes and the referential hollow cylinder marked by blue, red, green, and yellow curves, respectively.

Figure 11 shows the temporal signals received from the polygonal drill pipes and the referential hollow cylinder. In the figure, the first and second wave packets refer to the direct and reflected waves, respectively. It was obviously found that the time domain wave packets received from these waveguides were quite similar. With the increasing number of edges of the outer surface of the polygonal drill pipes, the time domain waveform was getting closer to the signal received from the referential hollow cylinder. These observations indicated that using ultrasonic guided waves for inspecting polygonal drill pipes was feasible. Furthermore, ultrasonic guided wave propagation characteristics from these polygonal drill pipes were quite similar. In the following sections, a specific polygonal drill pipe (a hexagonal drill pipe) was taken as an example to study ultrasonic guided waves propagating in normal and damaged polygonal drill pipes.

#### 4.1.3. Temporal Waveforms Received from Normal Hexagonal Drill Pipes with and without ALID Regions

In this subsection, temporal waveforms acquired from hexagonal drill pipes with and without applying ALID regions are compared. The effectiveness of ALID regions to remove the unwanted reflections from the boundaries is verified. The length of the hexagonal drill pipe *L* was set to 3200 mm. The excitation signal was a tone burst of 20 cycles at the center frequency of 60 kHz. Axial surface traction was enforced evenly and symmetrically around the excitation region to generated longitudinal guided modes. According to the dispersion curve presented in Figure 4c,d, both L(0,1) and L(0,2) modes would be generated. The parameters for the ALID regions were set as follows. The maximum attenuation parameter was set to 6 × 10^5^. The length of *L_a_* was 200 mm. The length of the sub layer *l_a_* was 4 mm, which was equal to element size. The value of *m* was set to 3.

**Figure 12:**
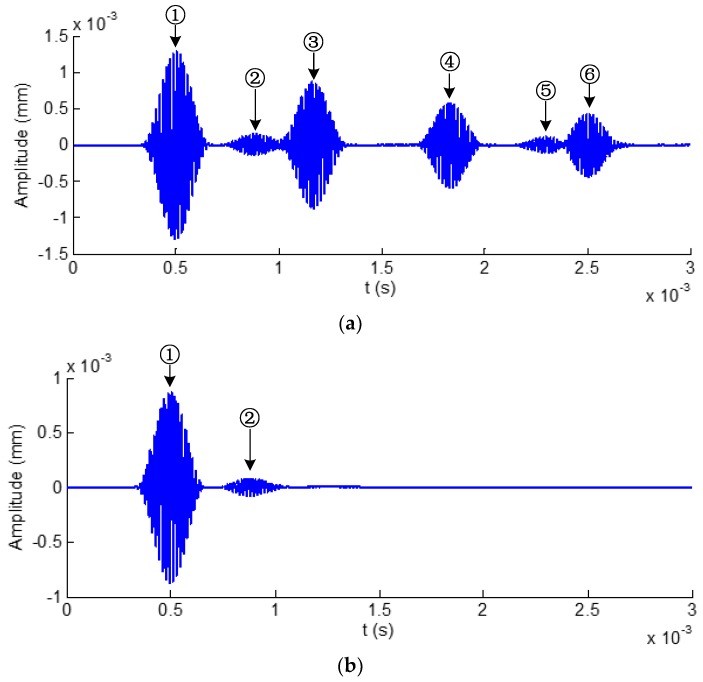
Time domain waveforms received from a hexagonal drill pipe: (**a**) Without and (**b**) with applying ALID regions.

**Figure 13:**
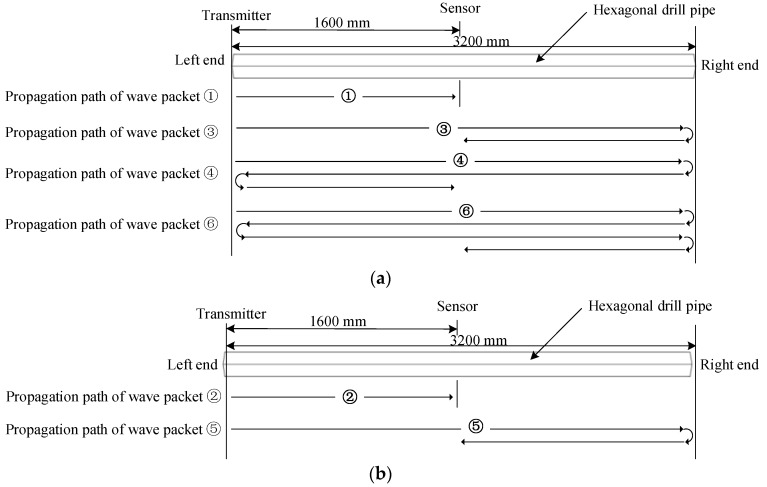
Wave propagation path analysis for each wave packet in the time domain waveform received from a hexagonal drill pipe without ALID regions: (**a**) L(0,2) mode; and (**b**) L(0,1) mode.

Time domain waveforms obtained from a hexagonal drill pipe without and with applying ALID regions are shown in Figure 12a,b, respectively. It is noted that these waveforms are acquired at the point that is located at the center of the hexagonal drill pipe. In Figure 12a, there are six wave packets which include the reflections from the left and right boundaries. They are divided into L (0,2) and L(0,1) modes. The wave packet ①, ③, ④, and ⑥ belong to L (0,2) mode. The rest wave packet ② and ⑤ are L(0,1) mode. The wave propagation path analysis for each wave packet are illustrated in Figure 13. Figure 13a,b show the analysis for wave packets of L(0,2) and L(0,1) modes, respectively. After applying ALID regions, from the Figure 12b, it was clearly observed that only the direct wave packets were present and all the reflections from the boundaries were eliminated. The wave packets ① and ② refers to the direct L(0,2) and L(0,1) modes, respectively. These results indicated the effectiveness of the ALID technique to eliminate the wanted boundary reflections. The using ALID facilitates the study of ultrasonic guided waves interacting with damages by avoiding overlapping the signals from a boundary and from a damage.

### 4.2. Ultrasonic Guided Waves Interacting with Damages in Polygonal Drill Pipes

In this section, ultrasonic guided waves of longitudinal and torsional modes interacting with symmetric and asymmetric damages in hexagonal drill pipes are studied in detail.

#### 4.2.1. L(0,1) Mode at the Center Frequency of 15 kHz

Axial surface traction was enforced uniformly and symmetrically around the excitation religion of a normal, symmetrically, and asymmetrically damaged hexagonal drill pipe, respectively. The excitation signal was a tone burst at the center frequency of 15 kHz with 10 cycles. According to the dispersion curve presented in Figure 4c,d, only the L(0,1) mode will be generated. The length of the hexagonal drill pipes *L* was 4800 mm. The symmetric and asymmetric damages were located 2000 mm from the left side. The length *L_w_* and height *L_h_* were set to 16 and 4 mm, respectively. ALID regions were applied to both sides of the hexagonal drill pipes. The parameters for the ALID regions were set as follows. The maximum attenuation parameter $\alpha_{\max}$ was set to 2 × 10^5^. The length of *L_a_* was 800 mm. The length of the sub layer *l_a_* was 4 mm, which was equal to element size. The value of *m* was set to 3.

**Figure 14:**
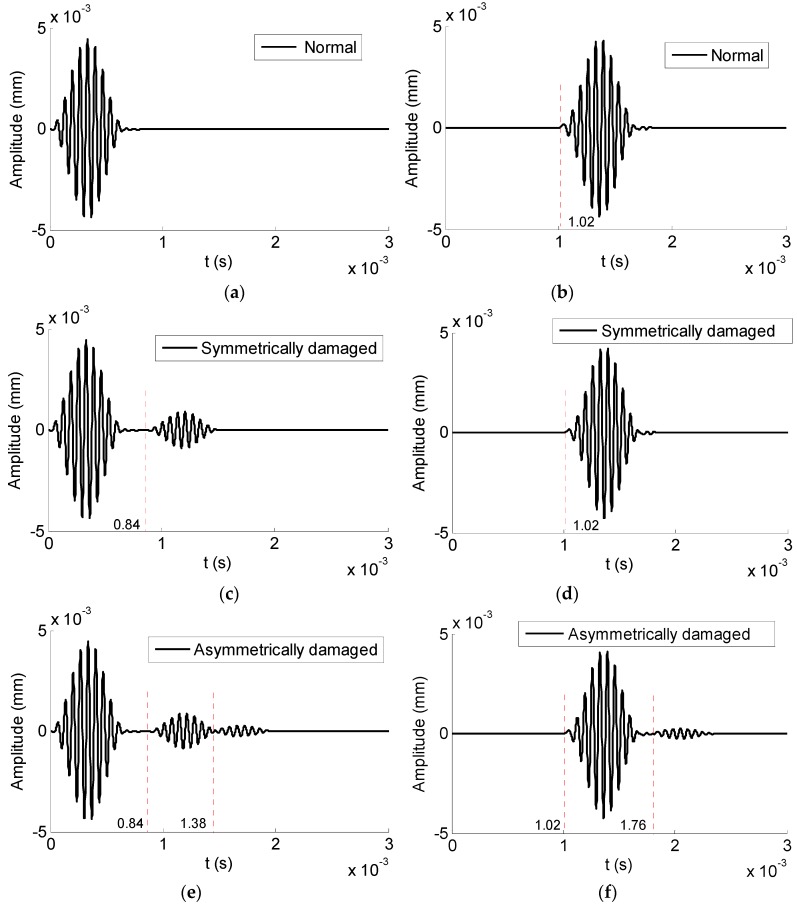
Time domain waveforms received from hexagonal drill pipes under the excitation of axial surface traction at the center frequency of 15 kHz: (**a**) and (**b**) reflected and transmitted waves from a normal hexagonal drill pipe, respectively; (**c**) and (**d**) reflected and transmitted waves from a symmetrically damaged hexagonal drill pipe, respectively; and (**e**) and (**f**) reflected and transmitted waves from an asymmetrically damaged drill pipe, respectively.

Reflected and transmitted time domain waveforms received from the normal hexagonal drill pipe are shown in Figure 14a,b, respectively. In the reflected temporal waveforms, there was no reflection. There was only wave packet and it was the excitation packet. The transmitted wave packet appeared at 1.02 × 10^−3^ s. Its propagation distance was 4800 mm. Therefore, the calculated group velocity was 4800 mm/1.02 × 10^−3^ s = 4706 m/s. It was close to the value 4656 m/s, which was the theoretical group velocity of L(0,1) mode at the frequency of 15 kHz, as shown in Figure 4d. It was verified that the wave packet is the direct L(0,1) mode.

Figure 14c,d illustrate the reflected and transmitted waveforms received from the corresponding symmetrically damaged hexagonal drill pipe. In the reflected waves, except for the excitation wave packet, there was one reflected wave packet. The wave packet appeared at 8.4 × 10^−4^ s. Suppose it was reflected from the damage, its propagation distance was 4000 mm. Therefore, the calculated group velocity of this wave packet was 4000 mm/8.4 × 10^−4^ s = 4762 m/s, which was also close to the theoretical group velocity value of L(0,1) mode at 15 kHz. It was verified that this wave packet was reflected from the symmetric defect. In the transmitted waves, the wave packet appeared at 1.02 × 10^−3^ s. It was the transmitted L(0,1) mode.

Reflected and transmitted waveforms from the asymmetrically damaged hexagonal drill pipe are shown in Figure 14e,f, respectively. In Figure 13e, except for the first excitation wave packet, there were two reflected wave packets in the domain waveform. The first wave packet appeared at 0.84 × 10^−3^ s. It had been verified that it was the reflected L(0,1) mode. The second one appeared at 1.38 × 10^−3^ s. Suppose it was converted from the L(0,1) mode due to the presence of the asymmetric damage. The calculated group velocity of the converted mode was 2000 mm/(1.38 × 10^−3^ − 0.84 × 10^−3^/2) s = 2170 m/s. It was close to 2232 m/s, which was the theoretical group velocity value of F(1,1) mode at 15 kHz, as illustrated in Figure 4d. It was proved that the second reflected wave packet was F(1,1) mode and it was converted from L(0,1) mode due to the presence of the asymmetric defect. In Figure 13f, the first wave packet was the transmitted L(0,1) mode. There was an additional wave packet. It appeared at 1.76 × 10^−3^ s. Suppose it was converted from L(0,1) mode, its calculated group velocity was 2800 mm/(1.76 × 10^−3^ − 0.84 × 10^−3^/2) s = 2121 m/s. It was close to the theoretical value. Therefore, it was verified that the second transmitted wave packet was F(1,1) mode, which was converted from the L(0,1) mode. From Figure 4d, it was found that at the frequency of 15 kHz, L(0,1) mode has the largest group velocity. The group velocity of the converted F(1,1) mode was smaller than that of L(0,1) mode. Therefore, in the reflected and transmitted waves, the converted F(1,1) wave packet laged behind the L(0,1) mode wave packet. The presence of the L(0,1) wave packet in the reflected time domain waveforms implied that there was a damage in the inspected hexagonal drill pipe. Furthermore, the presence of an additional converted wave packet in the reflected and transmitted waves indicated that the damage was asymmetric.

#### 4.2.2. Exciting Longitudinal Modes at the Center Frequency of 60 kHz

In the previous Section 4.2.1, axial surface traction was excited symmetrically at a low center frequency of 15 kHz to generate a single L(0,1) mode guided wave in hexagonal drill pipes. In this section, axial surface traction was excited at a slightly high center frequency of 60 kHz. According to the dispersion curve presented in Figure 4c,d, under the axial and symmetric excitation, L(0,1) and L(0,2) modes would be generated simultaneously. The excitation signal was a tone burst at the center frequency of 60 kHz with 20 cycles. The length of hexagonal drill pipes *L* was 6000 mm. The symmetric and asymmetric damages were located 2500 mm from the left side. The length *L_w_* and height *L_h_* were set to 8 and 4 mm, respectively. The parameters for the ALID regions were set as follows. The maximum attenuation parameter $\alpha_{\max}$ was set to 6 × 10^5^. The length of *L_a_* was 200 mm. The length of the sub layer *l_a_* was 4 mm, which was equal to element size. The value of *m* was set to 3.

**Figure 15:**
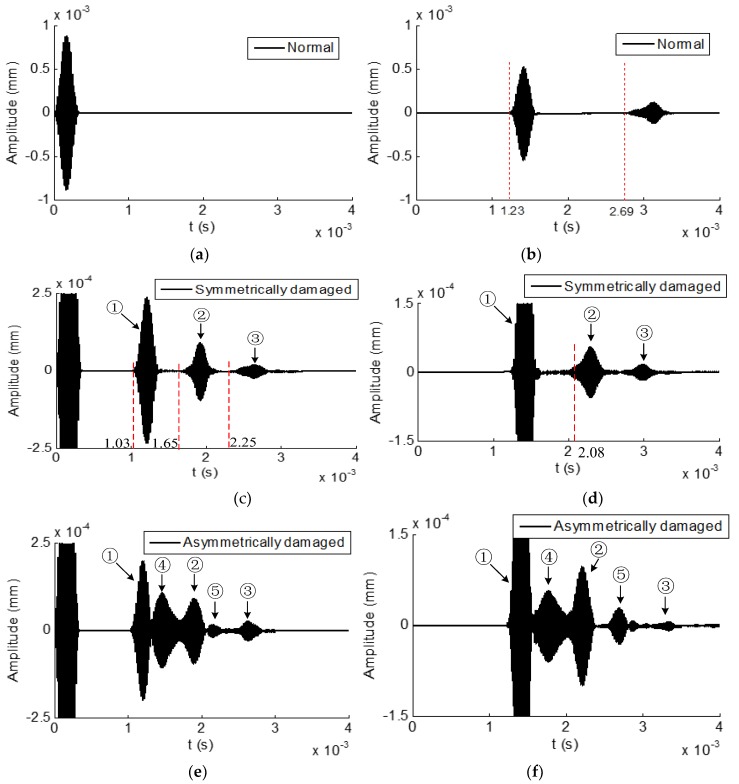
Time domain waveforms received from hexagonal drill pipes under the excitation of axial surface traction at the center frequency of 60 kHz: (**a**) and (**b**) reflected and transmitted waves from a normal hexagonal drill pipe, respectively; (**c**) and (**d**) reflected and transmitted waves from a symmetrically damaged hexagonal drill pipe, respectively; and (**e**) and (**f**) reflected and transmitted waves from an asymmetrically damaged drill pipe, respectively.

Reflected and transmitted time domain waveforms received from the normal hexagonal drill pipe are shown in Figure 15a,b, respectively. In the reflected temporal waveform, there was only the excitation wave packet. There were two wave packets in the transmitted time domain waveform. The first and second wave packets appeared at 1.23 × 10^−3^ s and 2.69 × 10^−3^ s, respectively. The propagation distance was 6000 mm. Therefore, the calculated group velocities were 6000 mm/1.23 × 10^−3^ = 4878 m/s and 6000 mm/2.69 × 10^−3^ s = 2230 m/s. They were close to the values 4893 and 2327 m/s, which were the theoretical group velocity of L(0,2) and L(0,1) modes at the frequency of 60 kHz, respectively. It was verified that the first and second wave packets were the direct L(0,2) and L(0,1) modes, respectively.

Figure 15c,d present the reflected and transmitted waveforms received from the corresponding symmetrically damaged hexagonal drill pipe, respectively. In the reflected waves, except for the excitation wave packet, there were three reflected wave packets. It could be verified that the wave packets ① and ③ referred to the reflected L(0,2) and L(0,1) modes, respectively. Furthermore, in the transmitted waves, the wave packets ① and ③ also denoted the transmitted L(0,2) and L(0,1) modes, respectively. How was the wave packet ② generated in the reflected and transmitted waves? Here the wave packet ② was analyzed. As the incident waves were the symmetric L(0,1) and L(0,2) modes and the damage was also symmetric, the wave packet ② could not be asymmetric or flexural modes. It could only be the symmetric modes. Exactly speaking, it could only be the longitudinal symmetric modes. According to the phase and group velocity dispersion curves in Figure 4c,d, at the frequency of 60 kHz, there are only two longitudinal symmetric modes, which are L(0,1) and L(0,2) modes. The wave packet ② propagated between the wave packets ① and ③. Its group velocity lies between the group velocities of L(0,1) and L(0,2) modes at the frequency of 60 kHz. Therefore, the wave packet ② was not the direct L(0,2) or L(0,1) mode. It could only be the converted mode. Suppose the wave packet ② was converted from L(0,2) to L(0,1) mode. In the reflected wave, the calculated propagation period was 2500 mm/4893 m/s + 2500 mm/2327 m/s = 1.59 × 10^−3^ s, which was close to the measured value 1.65 × 10^−3^ s. In the transmitted waves, the calculated propagation time was 2500 mm/4893 m/s + 3500 mm/2327 m/s = 2.02 × 10^−3^ s, which was also close to the measured value 2.08 × 10^−3^ s. It was thus verified that the wave packet ② was converted from L(0,2) mode to L(0,1) mode due to the presence of a symmetric damage. This finding was in accordance with the result obtained in a circular hollow cylinder with a symmetric defect reported in the previous study [26]. The mechanism of generating L(0,1) mode from the direct L(0,2) mode at the symmetric damage was not so clear. One possible reason is that the presence of the slot damage reduces the thickness of the polygonal drill pipe, which makes the excitation frequency bellow the cut-off frequency of the L(0,2) mode.

Reflected and transmitted time domain waveforms from the asymmetrically damaged hexagonal drill pipe is shown in Figure 15e,f, respectively. By comparing the Figure 14c,e, several findings were clearly observed. First, there were five wave packets in the reflected waves obtained from the asymmetrically damaged hexagonal drill pipe. Second, the arrival time of the wave packets ①, ②, and ③ in the reflected waveform from the asymmetric damaged hexagonal drill pipe were the same to the corresponding wave packets in the waveform from the symmetric damaged structure. The wave packets ① and ③ were the reflected L(0,2) and L(0,1) modes, respectively. The wave packet ② were the converted waves from the L(0,2) mode to L(0,1) mode due to the existence of the defect. Third, two additional wave packets, ④ and ⑤, appeared in the waveform, which was the converted flexural modes due to the asymmetric damage. The same findings could be obtained from the transmitted waves shown in Figure 15f. The presence of converted flexural modes in the reflected and transmitted waves can be used to identify the asymmetry of a damage in the hexagonal drill pipe.

#### 4.2.3. Exciting Torsional Mode at the Center Frequency of 50 kHz

In the previous Section 4.2.1 and Section 4.2.2, axial surface traction is excited to generate longitudinal modes in the hexagonal drill pipes. In this section, circumferential surface traction was excited symmetrically to generate a single T(0,1) mode guided wave in hexagonal drill pipes. The excitation signal was a tone burst at the center frequency of 50 kHz with 20 cycles. The length of hexagonal drill pipes *L* was 3000 mm. The symmetric and asymmetric damages were located 1000 mm from the left side. The length *L_w_* and height *L_h_* were set to 16 and 4 mm, respectively. The parameters for the ALID regions were selected as follows. The maximum attenuation parameter $\alpha_{\max}$ was set to 5 × 10^5^. The length of *L_a_* was 120 mm. The length of the sub layer *l_a_* was 4 mm, which was equal to element size. The value of *m* was set to 3.

**Figure 16:**
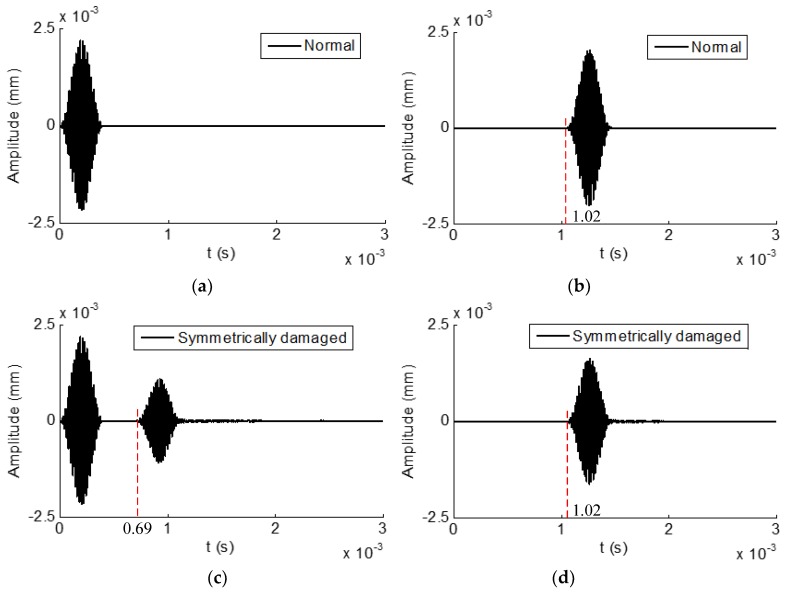

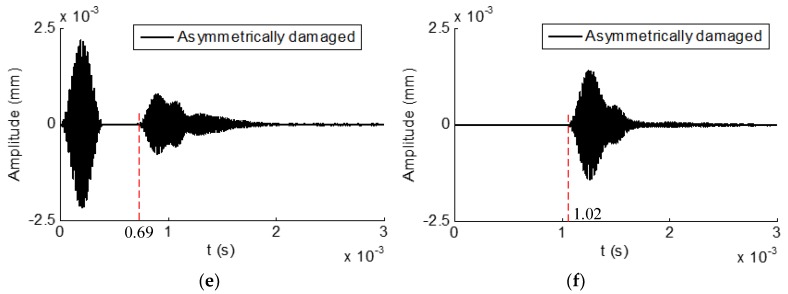
Time domain waveforms received from hexagonal drill pipes under the excitation of circumferential surface traction generating T(0,1) mode at the center frequency of 50 kHz: (**a**) and (**b**) reflected and transmitted waves from a normal hexagonal drill pipe, respectively; (**c**) and (**d**) reflected and transmitted waves from a symmetrically damaged hexagonal drill pipe, respectively; and (**e**) and (**f**) reflected and transmitted waves from an asymmetrically damaged drill pipe, respectively.

Torsional reflected and transmitted time domain waveforms received from the normal hexagonal drill pipe are shown in Figure 16a,b, respectively. In the reflected waves, only the excitation packet appeared. In the transmitted waves, the wave packet appeared at 1.04 × 10^−3^ s. Its propagation distance was 3000 mm. Therefore, the calculated group velocity was 3000 mm/1.04 × 10^−3^ s = 2884 m/s. It was close to the value of 2917 m/s which was the theoretical group velocity of T(0,1) mode at the frequency of 50 kHz, as shown in Figure 4d.

Figure 16c,d illustrate the torsional reflected and transmitted waveforms received from the corresponding symmetrically damaged hexagonal drill pipe. In the reflected waves, except for the excitation wave, the reflected wave packet appeared at 6.9 × 10^−4^ s. Suppose it was reflected from the damage, its propagation distance was 2000 mm. Therefore, the calculated group velocity of this wave packet was 2000 mm/6.9 × 10^−4^ s = 2898 m/s, which was also close to the theoretical group velocity value of T(0,1) mode at 15kHz. It was verified that this wave packet was reflected from the symmetric defect. In the transmitted waves, the wave packet appeared at 1.04 × 10^−3^ s. It was verified that it was the transmitted T(0,1) mode.

Torsional reflected and transmitted waveforms from the asymmetrically damaged hexagonal drill pipe are shown in Figure 16e,f, respectively. In Figure 16e, it was found that, except the excitation and the reflected T(0,1) wave packets, additional wave packets appeared in the reflected waves. They were converted flexural modes, which were due to the existence of an asymmetric damage. There were more than one converted modes in the waveform. The same observation could be found in the transmitted waves in Figure 16f.

## 5. Conclusions

In this paper, for the purpose of inspecting the long range of polygonal drill pipes by using the ultrasonic guided wave technique, characteristics of ultrasonic guided waves propagating in polygonal drill pipes are studied and the interactions between ultrasonic guided waves and damages are investigated. First, the phase velocity and group velocity dispersion curves in polygonal drill pipes are derived by using the semi-analytical finite element method. It is found that the multiple modes are in existence at a specific frequency and the number of modes is much larger than that of the referential hollow cylinder. As the number of edges of the outer surface of the cross section is increased, the phase and group velocity dispersion curves become sparse. Second, based on the derived phase velocity and group velocity dispersion curves, ultrasonic guided waves of longitudinal and torsional modes propagating in normal, symmetrically damaged, and asymmetrically damaged hexagonal drill pipes are studied. In order to eliminate the influence of boundary reflections, the ALID technique is applied to both ends of a hexagonal drill pipe to remove the unwanted end reflections. It is illustrated that, in the reflected waves, the presence of reflected wave packets implies the existence of damage in the inspected hexagonal drill pipe. Furthermore, the presence of converted flexural wave packets in the reflected or transmitted waves indicates that the damage is asymmetric. Our study results have shown that inspecting polygonal drill pipes using the technique of ultrasonic guided waves is feasible and effective. It is a promising method for detecting damage in polygonal drill pipes with high efficiency and accuracy. In future work, an experimental study on the inspection of polygonal drill pipes using ultrasonic guided waves will be conducted.
